# In-mask temperature and humidity can validate respirator wear-time and indicate lung health status

**DOI:** 10.1038/s41370-018-0089-y

**Published:** 2018-10-31

**Authors:** John W. Cherrie, Shuohui Wang, William Mueller, Charlotte Wendelboe-Nelson, Miranda Loh

**Affiliations:** 1 Heriot Watt University, Institute of Biological Chemistry, Biophysics and Bioengineering, Edinburgh, EH14 4AS UK; 2 Centre for Human Exposure Science, Institute of Occupational Medicine, Research Avenue North, Edinburgh, EH14 4AP UK

**Keywords:** Facemask, Respirator, Respiratory protective equipment, Exhaled breath temperature, EBT

## Abstract

The effectiveness of respiratory protection is dependent on many factors, including the duration and times during the day when it is worn. To date, these factors could only be assessed by direct observation of the respirator user. We describe the novel use of a data-logging temperature and humidity sensor (iButton Hygrochron) located inside a facemask to quantify respirator wear-time through supervised experiments (Phase 1) and an unsupervised wearing trial (Phase 2). Additionally, in Phase 1 the in-mask temperature was compared with measurements of exhaled breath temperature. We found humidity responds more rapidly than temperature to donning a mask, so it was considered a more sensitive measure of wear-time, particularly for short durations. Supervised tests showed that this method can provide accurate and precise estimates of wear-time, although the approach may be unsuitable for use in situations where there is high ambient humidity. In-mask temperature is closely associated with exhaled breath temperature, which is linked to lung inflammation. This technique could provide a useful way of evaluating the effectiveness of respirators in protecting health in real-life situations.

## Introduction

Respirators have been an integral part of protecting workers who may need to be in situations where the air around them contains high concentrations of hazardous pollutants. In addition, as the general public becomes more aware of the presence of and health impacts of air pollution, there is a demand for wearing respirators outside of the workplace, particularly in areas of high ambient pollution. Studies have found potential subclinical physiological responses of wearing respirators in high pollution situations, e.g., changes in ambulatory blood pressure and heart rate [1–3], although it has also been demonstrated that wearing a respirator may not provide consistent and effective protection and so the attribution of any physiological changes to reduced exposure is uncertain [4]. A simple method for effectively monitoring respirator wear is needed.

Temperature and humidity can easily be measured and data-logged by small, lightweight sensors. In theory, one can expect a rapid and high increase of these two variables relative to surrounding conditions upon donning of a respirator, which should last for the period of wear and decrease upon removal of respirator. Additionally, exhaled breath temperature (EBT) may provide some indication of lung inflammation. This study investigates the potential for a simple temperature and humidity logger to both assess wearer compliance and a potential health impact.

Respirators provide protection by filtering contaminants from the air before they are inhaled. The effectiveness of a device in reducing the inhaled contaminant is mainly dependant on the efficiency of the filter element and the effectiveness of the seal between the facepiece and the face, often called “edge seal leakage”. If a wearer removes the respirator or adjusts it over time such that it no longer provides a consistent seal, the effectiveness may be compromised; therefore, wear-time is also an important factor for the overall effectiveness of a respirator. However, this aspect can be more difficult to quantify than filter efficiency or leakage.

When used in workplaces, employers have a responsibility to train employees who are required to wear approved respirators to ensure they understand how to wear the device correctly. It is also their responsibility to supervise the wearing of respirators, although it is generally impractical for this to be done continuously. Respirators are now also being used by consumers to provide protection against particulate air pollution or dusts generated during do-it-yourself (DIY) activities. In these circumstances, with a much wider range of equipment in use, there is generally no requirement to provide approved devices, and there is unlikely to be any appropriate training or supervision of consumer wearers.

Recent research examined the effectiveness of a range of masks available to consumers [4]. Temperature measured inside the facemasks during these tests did not show any clear association with the measured effectiveness of the respirator in the test. When respirators are correctly fitted, the temperature and humidity inside the facepiece should reflect those of exhaled breath, which, depending on the setting, may be distinct from ambient levels. These changes occur because air rebreathed into the mask has been heated and humidified in the lungs. Continuously monitoring the temperature and humidity inside the mask may therefore provide a means to assess when, and for how long, a mask is worn.

In addition to validating the wear-time of facemasks, EBT and humidity are associated with lung inflammation [5, 6]. It is argued that increased inflammation is linked to increased blood flow in the lung airway walls and, consequently, greater heat transmission to the surrounding air [7]. These authors demonstrated that EBT was significantly higher in asthmatics than in control subjects, and that in a group of asthmatic children there was a significant positive association between EBT and both exhaled nitric oxide and eosinophil percentage in induced sputum. Additionally, Popov and colleagues showed that there was a significant reduction in EBT after treatment with corticosteroid medication in adult asthmatics [8]. EBT has been investigated as a tool for managing asthma symptoms [9,10] to help diagnose COPD in smokers [11]. There is limited research on differences in exhaled humidity associated with asthma.

In this paper, we present two experiments that evaluate the use of a simple data-logging temperature and humidity sensor to (1) validate respirator wear-time, (2) compare in-mask temperature to EBT measured using a commercial clinical measurement system and (3) to assess EBT as a measure of lung health.

## Methods

A Hydrochron iButton® DS1923 (Maxim Integrated, San Jose, CA, USA) was attached with adhesive inside a disposable filtering facepiece respirator. The iButton contains a battery and can log temperature and relative humidity (RH) at regular intervals (in this case each 10-s) to a resolution of 0.5 °C and 0.6% RH. The measurement ranges are −20 to +85 °C and 0 to 100% RH. EBT whilst not wearing a mask was measured with an X-halo EBT monitor (http://www.x-halo.com/home/).

**Fig. 1:**
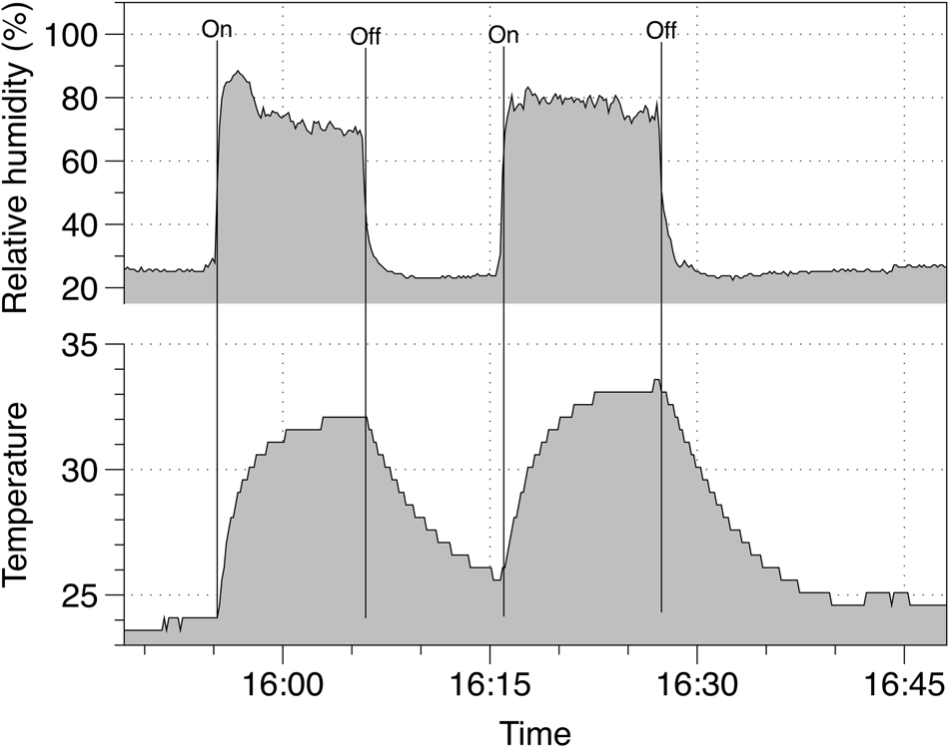
Typical temperature and humidity profile from donning a respirator on two occasions—on/off times as recorded by the participant

In Phase 1, to test the reliability of the iButton data to indicate wear-time, subjects put on and removed on two occasions for about 10 min each a disposable filtering facepiece respirator (3M 8210) with an iButton sensor inside. Separate EBT measurements were taken either before or after each of the iButton trials. The start and stop wear-times were recorded and compared with the iButton logged temperature and humidity data; the wear-times were identified by RH > 75% and modelled steady-state temperature > 27 °C (see Data Analysis section). Figure 1 shows example data to illustrate changes to temperature and humidity whilst wearing a respirator for 10-min periods. The order of completing the test was alternated between subjects, i.e., mask wearing, X-halo EBT measurement, with the sequence repeated or X-halo EBT measurement, mask wearing, then repeated. All tests were carried out indoors in either an office or home environment, and wearers were given no special instructions about how to fit or wear the respirator. The study involved adults.

In Phase 2, a pilot field trial, adult volunteers (who had not participated in Phase 1) were recruited from central Scotland to wear a disposable respirator (3M 9211) with an in-mask iButton sensor during their daily activities to protect against particulate air pollution. Individuals were primarily recruited through a citizen-scientist activist and from the local student population. Each participant was asked to wear the respirator wherever and whenever they felt it would be appropriate to protect their health during normal daily activities over 5 days. As in the first phase of the work, the wearers were given no special instruction about fitting or wearing of the masks. Temperature and humidity were logged by the iButton every minute to permit the extended duration of monitoring; this longer interval length precluded more complex modelling to estimate the equilibrium in-mask temperature, and in this case we relied upon the measured temperature and humidity data. Participants were asked to keep a brief diary of their mask usage, i.e., time when the mask was put on and taken off, and to record any observations about the mask when wearing it, e.g., comfort, effectiveness, perceived changes to respiratory symptoms. Data from the iButton sensors were downloaded at the end of the 5-day period. Subjects also completed a short questionnaire about their respiratory health status, smoking habits and demographic data.

Ethical approval was obtained from Heriot Watt University Engineering and Physical Science Ethics Committee. The approval numbers were 17/EA/JC/2 and 17/EA/JC/3. Written informed consent was obtained from each participant.

### Data analysis

For Phase 1, because of the relatively short wearing time, the in-mask temperature was estimated by fitting an equation of the form to the temperature (*T*) and time (*t*) data using the R statistical function “nls”:

$$T = a.\left( {1 - e^{k.t}} \right)$$

where the estimate of the maximum steady-state temperature inside the mask (*T*_max_) is:

$$T_{{\mathrm{max}}} = T_{{\mathrm{base}}} + a$$

and *T*_base_ is the baseline temperature prior to the mask being donned. The terms ‘*a*’ and ‘*k*’ are constants fitted in the analysis.

For Phase 2, the mean temperature and RH less one standard deviation were used as criteria to identify a threshold upon which to objectively assess when the respirator was worn. These values were based on times in the diary when wearing the mask was indicated. This was assumed to be reasonable since volunteers would not likely record wearing the mask if they were not actually doing so, though they very well may do the opposite, i.e., forget to record wearing it. The agreement of wear-time between the mask and diary was assessed using Spearman correlation, as well as sensitivity and specificity. In addition, as volunteers may be apt to estimate when they started and stopped wearing the mask, e.g., rounding to the nearest 5 min, such patterns of the start/stop times of the diary and iButton were examined using a Chi-squared test.

A regression model was developed to assess the effects of asthma, age and gender on temperature, whilst wearing a respirator. To adjust for serial autocorrelation, which may occur during repeated measurements over a period of time, a Cochrane–Orcutt regression model was applied. The Durban–Watson statistic was adjusted from 0.10 to 1.14, which is an improvement, though still suggests the presence of some residual correlation [12]. The statistical programmes R (R development core team) and Stata v.15 (Statacorp, College Station, TX, USA) were used for statistical analysis.

## Results

**Fig. 2:**
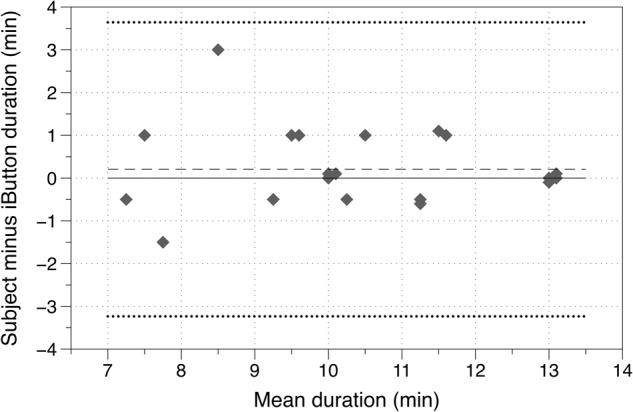
Bland–Altman plot comparing duration of wear recorded by the subject and from iButton data (the dashed line shows the mean difference in duration, and the dotted lines the limits of agreement)

In Phase 1, 11 people (six men and five women) participated in the initial tests to validate the reliability of the iButton to assess wear-time and EBT. Figure 2 compares the wear-time duration recorded by each subject and the wear-time derived from the iButton data, on two occasions, in a Bland–Altman plot. The plot shows the mean of the two measures and the difference between the duration assessed by the subject and the iButton data. The mean wearing duration ranged from 7.25 to 13.0 min. There is little evidence of any systematic difference between the two measures; for all but one of the subjects, the recorded and estimated times agreed within approximately ±1 min.

**Fig. 3:**
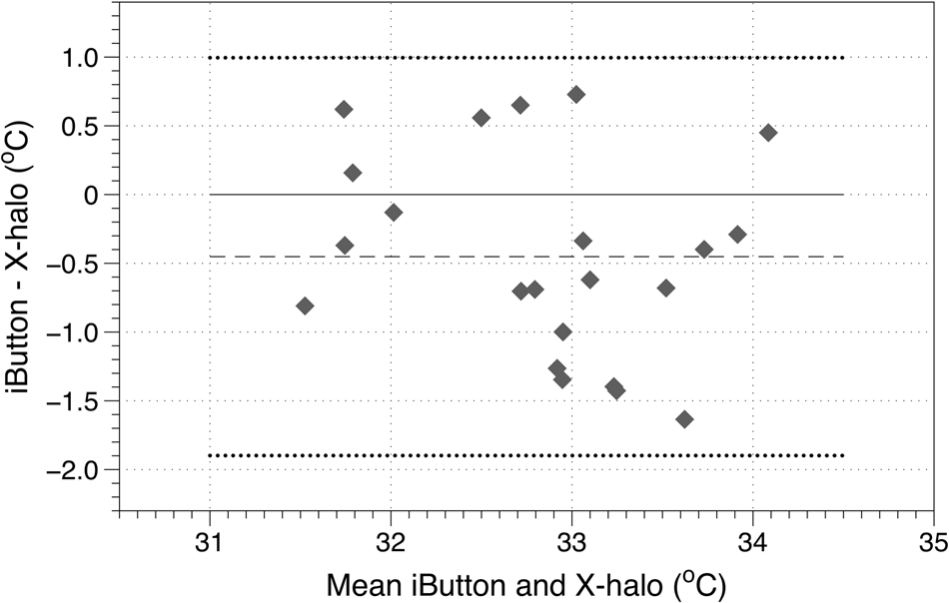
Bland–Altman plot of mask and X-halo temperatures (dashed line shows the mean difference in temperature, and the dotted lines the limits of agreement)

Figure 3 compares the in-mask temperature measured using the iButton (steady-state estimate) with the EBT measured using the X-halo instrument. The mean temperature ranged from 31.5 to 34.1 °C, and the difference in temperature between the two measures ranged from −1.64 to 0.73 °C. The mean difference between the iButton and the X-halo temperatures was −0.45 °C; this was significantly different from zero in a Shapiro–Wilk test, *p* = 0.02.

In the pilot field trial (Phase 2), nine (from 18) of the volunteers who wore the respirators recorded in a diary when they did so. Six volunteers were aged over 65 years, five were male, and two reported they were asthmatic (both males). An example temperature and humidity record from one of the participants is shown as Supplementary Figure 1.

The calculated thresholds for temperature and RH, using rounded values, were 25 °C and 77% RH, respectively (Table 1). Using the iButton data, the longest duration that a given volunteer continuously wore a respirator varied widely, ranging from 9 min to nearly 3 h.

**Table 1:** The temperature and relative humidity (RH) across all volunteers when the mask was recorded in the diary as on or off

Mask	Mean	SD	25th Pctl.	50th Pctl.	75th Pctl.
	Temperature (°C)				
On	29.2	3.60	28.2	30.2	32.1
Off	21.5	2.90	19.7	21.1	22.7
	Relative humidity (%)				
On	88.5	11.7	85.9	91.3	95.5
Off	61.6	11.2	54.5	62.0	67.4

There was a very high rate of agreement between the diary and the values indicated by the iButton thresholds (Supplementary Table 1). The Spearman rank coefficient between these two variables was 0.87 (*p* < 0.001). The sensitivity of the diary compared to the iButton was 92.5% and the specificity was 98.8%.

Of the 46 start times reported in the diary, 42 (91.3%) occurred on a time divisible by 5 min (*Χ*^2^ = 52.5; *p* < 0.001). Similarly, for the stop times, 33 of 46 (71.7%) were recorded on such a time (*Χ*^2^ = 48.3; *p* < 0.001). By contrast, iButton times did not suggest any pattern for either the start (*Χ*^2^ = 9.2; *p* = 0.417) or stop (*Χ*^2^ = 7.8; *p* = 0.559) times.

The regression model suggested that having asthma was found to be significantly related to higher EBT whilst wearing a respirator (*p* < 0.001). Conversely, being over the age of 65 years was associated with lower EBT (*p* = 0.006). Gender was not a statistically significant explanatory factor in this pilot study (*p* = 0.325; Table 2).

**Table 2:** The coefficients (°C) and 95% confidence intervals of effects on exhaled breath temperature whilst wearing a respirator

Variable	Coefficient	95% Confidence v	*p*-value
Asthma	1.71	1.23 to 2.19	< 0.001
> 65 years of age	−1.61	−2.75 to −0.46	0.006
Male	−0.34	−1.01 to 0.33	0.325
Constant (°C)	32.4	31.4 to 33.4	–

*N* = 2379; R-squared = 0.02

Participants who wore the respirator provided qualitative feedback. Several reported being self-conscious when wearing the mask in public, especially the female participants. All of the female participants who completed the questionnaire reported that strangers in public commented on the mask, causing them to feel self-conscious and embarrassed. Participants also reported feeling discomfort from wearing the mask, and those who wore spectacles described how they could “mist-up” when wearing the facemask. Most of the participants felt sweaty after wearing the facemask, and some wrote that the facemask had an “unusual” smell. A few subjects wrote that they felt breathlessness after wearing the facemasks, including one participant who had a history of asthma. One participant reported having a headache after wearing the facemask.

## Discussion

From the experiments we have carried out, we show the iButton can provide an accurate tool to track wear-time to within about a minute of the time recorded by a subject. We have also shown that in practice many subjects were not motivated to keep a diary record of when they wore a facemask during their daily activities, so iButton data can overcome this obstacle. When individuals did keep a diary record of when they wore the facemask, the data agreed well with those from the iButton. Another advantage presented by the iButton is that it may provide start and stop wear-times that are more precise than those recorded by volunteers. The diary times appeared to be often rounded to the nearest 5 min, whilst the iButton data did not demonstrate this pattern. From the results of our pilot study, we believe that this type of measurement device is an essential part of any attempt to quantify the actual effectiveness of respirators in protecting individuals who have pre-existing cardiovascular or respiratory disease from particulate air pollution. Nevertheless, the iButton does have limitations in its use; for example, it would be unsuitable for use in environments where there are high ambient humidity and temperature because the method requires a contrast between the environment and inside the facepiece while being worn. It would also be unsuitable for masks that are flat to the face, e.g., a surgical mask, because of the likely contact of the sensor with the skin. The iButton also takes around 10 min to completely equilibrate to the environmental temperature because of the inherent thermal inertia of the sensor package (mass 3.1 g).

In previous research we found no indication that in-mask temperature was associated with the effectiveness of the mask, although in those tests we did not include humidity measurements [4]. It is most likely that the temperature and humidity inside a facepiece are predominantly determined by the exhaled breath rather than inhaled air. In assessing respirator effectiveness, the iButton is therefore only suited to assess wear-time not inward leakage of pollutants. This could be used as part of a workplace respirator programme where the employer would use the iButton to ensure that respirators, which were used by trained fit-tested wearers, were being worn appropriately, or in a research study in the community to investigate the effects of wearing a facemask on respiratory or cardiac biomarkers, where knowing when and where the mask was worn would be necessary information.

In addition to the comparison of mask wear-times, we also identified slightly, but significantly, lower in-mask temperatures as measured by the iButton, when compared to out-of-mask EBT measurements obtained from the X-halo device. Such discrepancies may result from variations in the accuracy of these devices, or more probably because the in-mask measurement reflects the inhalation and exhalation breathing cycle rather than just the exhaled temperature. A number of alternative methods of measuring EBT have been proposed, including commercialised instruments: closed-circuit multi-breath and open-circuit devices [13], though there are no data to determine how the measurement method affects the measured EBT.

In-mask temperature may be a useful biomarker to assess the exacerbation of disease from exposure to particulate air pollution. Despite the observed discrepancy between in-mask temperature and out-of-mask EBT, we identified a statistically significantly increased in-mask temperature for the self-reported asthma subjects compared to the others in the field trial. However, other than asthma status, these increases could also be due to other endogenous or environmental factors that may affect in-mask temperature and that were unaccounted for in our study. For example, EBT is increased within 1 h of consuming food [13], whilst smokers who give up smoking show a steady decline in EBT over a period of several weeks [14]. Bijnens et al. showed that EBT was associated with the proximity of the residence of the subject to a major road, with increasing traffic density within 500 m of the home, and inversely associated with age [15]. Svensson and colleagues investigated EBT in asthmatics and healthy controls after exercise [16]. In their tests, the median EBT increased after exercise with no significant difference between the groups. In a larger study, it may be possible to take into account some of these other factors, whilst at the same time identifying variation in in-mask temperature, and possibly humidity, in relation to the extent of exposure to pollution.

Our pilot studies have demonstrated that the use of in-mask temperature and humidity measurement offers a simple low-cost way of characterising respirator use, and simple biomarkers of lung inflammation. The iButton would appear to offer a simple and reliable way to record this type of data over extended periods, up to a week. The approach is a valuable technique that should be used in assessing the benefit to health from wearing respirators. There are a number of research studies that have demonstrated the health benefits of wearing a facemask in a controlled test environment, and the next step could be to use the iButton to help identify whether wearing a mask in a realistic non-prescriptive way in the community has similar benefits in reducing the effects of particulate air pollution.

## Electronic supplementary material

Supplementary Figure

Supplementary Information
