# Supplementary figures and images for: In-mask temperature and humidity can validate respirator wear-time and indicate lung health status
Source: J Expo Sci Environ Epidemiol. 2018 Oct 31;29(4):578–83. doi: 10.1038/s41370-018-0089-y (PMC6760615; doi:10.1038/s41370-018-0089-y)

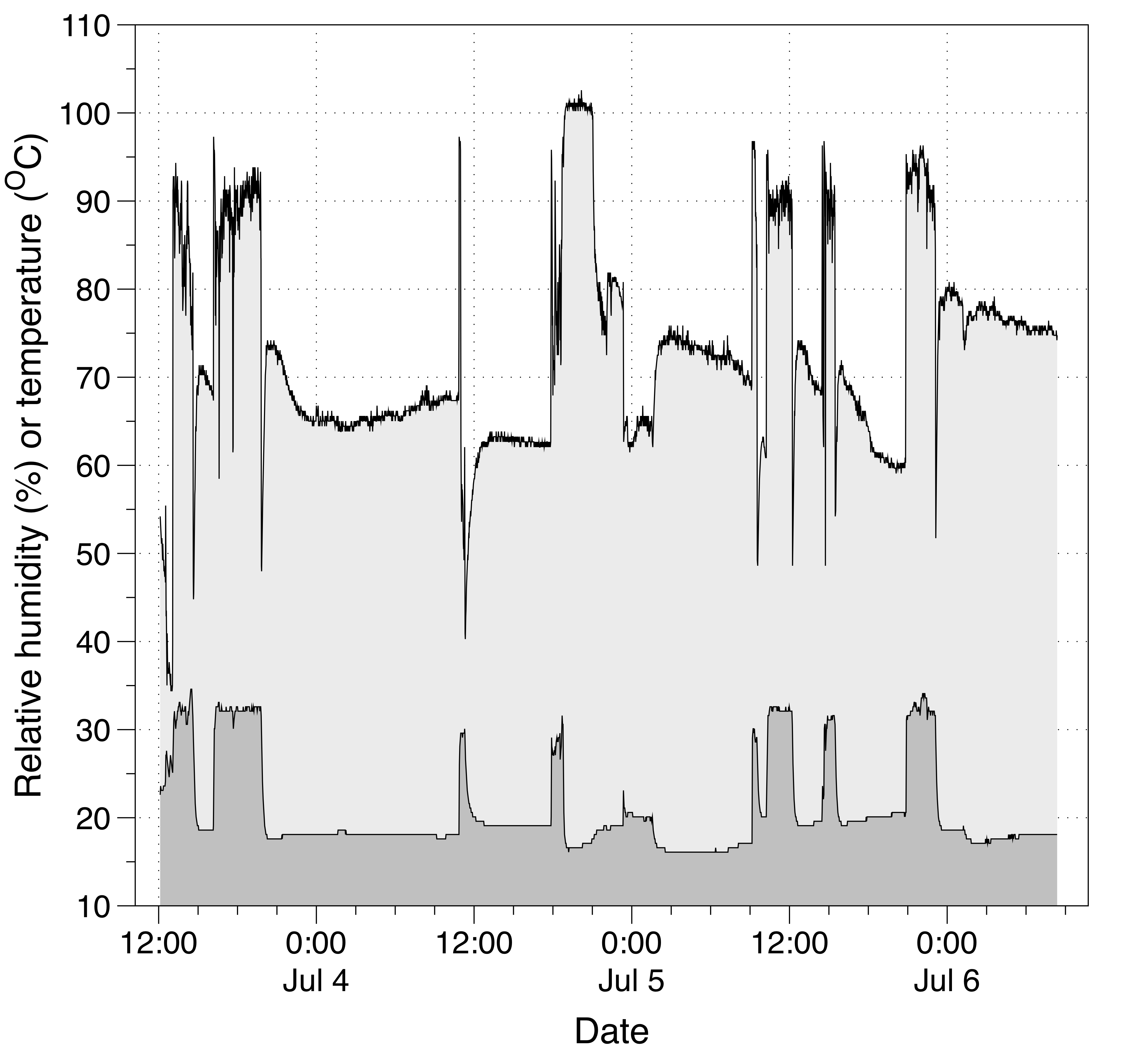

Supplement: Supplementary file 1 — Supplementary Figure [file 41370_2018_89_MOESM1_ESM.tiff]
