# Supplementary material for: In-mask temperature and humidity can validate respirator wear-time and indicate lung health status
Source: J Expo Sci Environ Epidemiol. 2018 Oct 31;29(4):578–83. doi: 10.1038/s41370-018-0089-y (PMC6760615; doi:10.1038/s41370-018-0089-y)
Supplement: Supplementary file 2 — Supplementary Information [file 41370_2018_89_MOESM2_ESM.docx]

**ON LINE MATERIAL**

**Supplementary Table 1:** The agreement between the diary and iButton: number of observations and (%).

|  | **iButton** | |
| --- | --- | --- |
| **Diary** | **On** | **Off** |
| **On** | 2,276(6.2) | 426 (1.2) |
| **Off** | 185 (0.5) | 33,977 (92.2) |

**Supplementary Figure 1:** Example of temperature and humidity data log for one participant over 4-days
